# Systemic effects of angiogenesis inhibition alter pharmacokinetics and intratumoral delivery of *nab*-paclitaxel

**DOI:** 10.1080/10717544.2017.1406559

**Published:** 2017-11-24

**Authors:** Anne Steins, Eva A. Ebbing, Marcel C. M. Pistorius, Cynthia Waasdorp, Kausilia K. Krishnadath, Jan Paul Medema, Johanna W. Wilmink, Ron A. A. Mathôt, Maarten F. Bijlsma, Hanneke W. M. van Laarhoven

**Affiliations:** a Cancer Center Amsterdam, Laboratory for Experimental Oncology and Radiobiology, Center for Experimental and Molecular Medicine, Academic Medical Center, Amsterdam, The Netherlands;; b Department of Medical Oncology, Academic Medical Center, Amsterdam, The Netherlands;; c Department of Hospital Pharmacy, Academic Medical Center, Amsterdam, The Netherlands;; d Department of Gastroenterology and Hepatology, Academic Medical Center, Amsterdam, The Netherlands;; e Cancer Genomics Center, Center for Molecular Medicine, Utrecht, The Netherlands

**Keywords:** Angiogenesis, anti-angiogenic therapy, esophagogastric adenocarcinoma, nab-paclitaxel, pharmacokinetics

## Abstract

Angiogenesis is critical to the growth of tumors. Vascularization-targeting agents, with or without cytotoxic drugs, are widely used for the treatment of several solid tumors including esophagogastric adenocarcinoma. However, little is known about the systemic effects of anti-angiogenic therapies and how this affects the pharmacokinetics and intratumoral delivery of cytotoxic agents. In this study, patient-derived xenograft mouse models of esophageal adenocarcinoma were used to identify the effects of DC101, a murine vascular endothelial growth factor receptor 2 (VEGFR2) inhibitor, on the pharmacokinetics and the intratumoral uptake of *nab*-paclitaxel (NPTX). We showed that DC101 had large systemic effects resulting in decreased vasculature of intraperitoneally located organs. As a consequence, after intraperitoneal administration of NPTX, plasma uptake (5.029 ± 4.35 vs. 25.85 ± 2.27 µM) and intratumoral delivery (5.48 ± 5.32 vs. 38.49 ± 2.805 pmol/mg) of NPTX were greatly impaired in DC101-treated animals compared to control animals. Additionally, routes of NPTX elimination were altered upon angiogenesis inhibition; unchanged renal clearance and intraperitoneal accumulation of NPTX were observed, but NPTX levels were significantly lower in the liver. Histological examination of the intestine revealed a reduced thickness of the intestinal wall following DC101 therapy and suggested seepage of intraperitoneally injected NTPX through the intestinal wall to explain its reduced uptake in liver, plasma, and tumor tissue. These data explain several adverse effects observed in the clinic when using anti-angiogenic therapies and also imply that the combined use of anti-angiogenesis and cytotoxic agents in both preclinical and clinical setting is still suboptimal.

## Introduction

Angiogenesis is critical to the growth of tumors and it has been known for long that inhibition of neovascularization could suppress tumor growth (Sherwood et al., 1971). Vascular endothelial growth factor (VEGF) and its receptor vascular endothelial growth factor receptor 2 (VEGFR2) are essential for the formation of new vessels. Most solid cancers overexpress these proteins and aberrant VEGFR2 regulation appears to be related to poor prognosis and therapy resistance in various malignancies including lung, gastric and breast cancer (Padró et al., 2002; Rydén et al., 2005; Carrillo de Santa Pau et al., 2009; Hirashima et al., 2009; Carmeliet & Jain, 2011; Kampen, 2012). Ramucirumab (Cyramza), a humanized monoclonal antibody against the extracellular domain of VEGFR2, inhibits downstream signaling by preventing receptor dimerization and intracellular autophosphorylation (Clarke & Hurwitz, 2013). Ramucirumab has been approved by the US Food and Drug Administration (FDA) for the treatment of several solid tumors including advanced esophagogastric adenocarcinoma (AEG) (Fuchs et al., 2014; Garon et al., 2014; Wilke et al., 2014; Young et al., 2015; Roviello et al., 2017). However, survival benefits of monotherapy are only marginal and anti-angiogenic drugs are therefore usually given in combination with conventional cytotoxic drugs, such as ramucirumab and paclitaxel in the treatment of esophagogastric junction tumors (Ma & Waxman, 2008; Young et al., 2015).

Although the effects of anti-angiogenic therapies on the vasculature of tumor tissue have been widely studied, only a few studies describe the off-target systemic effects in tumor-free healthy tissue. In preclinical mouse models these studies demonstrated that not only tumor vessels, but also normal blood vessels depend on VEGF signaling. Neutralizing antibodies against both VEGF and VEGFR2 resulted in robust vascular regression in organs such as the pancreas, uterus, thyroid gland, and small intestine and this was accompanied by loss of functionality of some of these organs (Kamba, 2005; Yang et al., 2013).

Intraperitoneal (i.p.) administration of agents is a commonly used method in preclinical studies, as intravenous access is challenging in small rodents and larger volumes of fluid can be administered (Turner et al., 2011). Also in clinic, techniques such as Hyperthermic Intraperitoneal Chemotherapy (HIPEC) use the advantages of i.p. administration to treat peritoneal dissemination of gastrointestinal cancer (González-Moreno et al., 2010). Based on the described systemic vascular effects, we hypothesized that the pharmacokinetics and systemic uptake of i.p. administered chemotherapy could be affected when given in combination with anti-angiogenic therapy. In the present study, patient-derived xenograft mouse models of esophageal adenocarcinoma (EAC) were used to identify the effects of DC101, a murine VEGFR2 inhibitor, on pharmacokinetics and intratumoral uptake of i.p. administered *nab*-paclitaxel (NPTX) and aimed to elucidate the mechanisms by which these changes occur.

## Materials and methods

### Establishment of primary cultures

Tumor material of patients diagnosed with EAC in the Academic Medical Center (Amsterdam, the Netherlands) was collected as described earlier and approved by the institute’s ethical committee and performed according to the guidelines of the Helsinki Convention (MEC 01/288#08.17.1042) (Damhofer et al., 2015). Patient material was expanded by subcutaneous xenograft in immunocompromised NOD.Cg-*Prkdc^scid^ Il2rg^tm1Wjl^*/SzJ (NSG) mice, which were bred and maintained at the local animal facility according to the legislation and ethical approval by the animal experiment ethical committee (LEX100780 and LEX102774). Primary cultures were established as described earlier (Damhofer et al., 2015). The primary culture used for these studies is AMC-EAC-007B (007B), a pretreatment biopsy, and cells were cultured according to standard culture procedures in Iscove’s Modified Dulbecco’s Medium supplemented with 8% fetal calf serum, L-glutamine (2 mmol/l), penicillin (100 units/ml), and streptomycin (500 µg/ml; Lonza, Basel, Switzerland).

### Reagents

DC101, a generous gift from Imclone Systems (Eli Lilly and Company, Indianapolis, IN) was reconstituted in phosphate-buffered saline (PBS). *Nab*-paclitaxel (NPTX, Abraxane®, Celgene, Summit, NJ), a new formulation of paclitaxel, was purchased from our hospital pharmacy and reconstituted in normal saline, prepared fresh, and administered within 2 h after preparation (Schaumburg, 2005). The control treatment for NPTX was normal saline and the control treatment for DC101 was PBS (Fresenius Krabi, Bad Homburg, Germany).

### Animals and tumor inoculation

Female athymic nude Foxn1^nu^ mice which were 6 weeks of age and 20–25 g in weight were purchased from Envigo. All animal experiments were performed in the local animal facility and in accordance with the legislation and ethical approval by the animal experiment ethical committee (LEX103159). Mice were anesthesized using 3% isoflurane, and subcutaneously injected on the right hind limb with 1 × 10^6^ early passage 007B cells suspended in 200 µl PBS. Tumor growth, weight, and welfare were measured twice a week.

### Treatment schedule and sample collection

For pharmacokinetic studies of NPTX in different tissues, tumor-bearing mice (∼800 mm^3^) were randomly divided into five groups and received a single bolus of 120 mg/kg of body weight NPTX, the reported maximum well-tolerated dose, or saline control *via* i.p. injection (Desai et al., 2008; Neesse et al., 2014). At 1, 2, 4, and 24 h post-injection, mice were anesthetized using isoflurane and sacrificed by heart puncture. Blood was collected in BD Vacutainer^®^ K2 EDTA blood collection tubes which were spun down at 1300 RCF for 10 min and plasma was stored at −80 °C. The following tissues were harvested, snap frozen, and stored at −80 °C until further processing for liquid chromatography/tandem mass spectrometry (LC–MS/MS); intestine, liver, kidney, spleen, pancreas, tumor, lung, fat, muscle, skin, and brain.

For intratumoral NPTX uptake studies after DC101 therapy, tumor bearing mice (50–75 mm^3^) were randomly divided into two groups and injected with either DC101 (40 mg/kg) or PBS *via* i.p. injection. Both therapies were given twice a week for four weeks, or until tumor volume had reached the maximum allowed size of 1000 mm^3^. Three days after the last injection of DC101 or PBS, a single bolus of 120 mg/kg of body weight NPTX was given *via* i.p. injection. Two hours after NPTX injection, mice were anesthetized and blood was collected and processed as described earlier. Tumor tissue was harvested, snap frozen, and stored at −80 °C until further processing for LC–MS/MS. The following tissues were harvested and fixated in 4% paraformaldehyde (PFA); peritoneum, liver, kidney, intestine, and tumor.

To study the routes of NPTX elimination, mice were randomly divided into two groups and injected i.p. twice a week for four weeks with either DC101 (40 mg/kg of body weight) or PBS. Three days after the last injection of DC101 or PBS, a single bolus of 120 mg/kg NPTX was given *via* i.p. injection. Two hours later, mice were anesthetized and any remaining NPTX in the peritoneal cavity was harvested by i.p. lavage with 2.5 ml of cold saline (i.p. flush). Subsequently, mice were sacrificed and urine was collected and, together with i.p. flush samples, stored at −80 °C. Liver and kidney were harvested, snap frozen, and stored at −80 °C until analysis by LC–MS/MS.

### Liquid chromatography/tandem mass spectrometry

Tissue samples which were snap frozen and stored at −80 °C were weighed and diluted in normal saline (0.1 mg/ml). Samples were homogenized using the TissueLyser LT (Qiagen, Hilden, Germany) for 3–6 min at 50 Hz. Plasma, i.p. flush, and urine samples could be used directly for analysis. Paclitaxel concentrations were determined using an LC–MS/MS method validated according to the principles of the FDA guidelines. After protein precipitation, samples were analyzed using LC–MS/MS. Calculation of the concentration was performed *via* a standard dilution series of paclitaxel in human plasma using the internal standard method. The LC–MS/MS setup consisted of a LC-30 Nexera (Shimadzu, Kyoto, Japan) system coupled to an API 5500 Mass spectrometer system (AB Sciex, Concord, ON, Canada). Mass transition of paclitaxel precursor-ion was 854.35 *m*/*z* and product-ion was 569.25 *m*/*z*. Mass transition of ^13^C_6_-Paclitaxel precursor-ion was 860.35 *m*/*z* and product-ion was 575.25 *m*/*z*. The lower and upper limits of quantification were 1–250,000 ng ml^−1^ for paclitaxel.

### Immunohistochemistry

After overnight fixation in 4% PFA, tissues were dehydrated in a series of ethanol. After overnight incubation at 65 °C in paraplast tissues were embedded in paraffin and 4 µm thick sections were cut on a microtome. Tissue sections were deparaffinized and rehydrated in a series of ethanol. Heat-mediated antigen retrieval was performed using Tris-EDTA buffer solution at pH 9 (Lab Vision™ PT Module™, Thermo Scientific, Waltham, MA). Endogenous peroxidase was blocked using 3% hydrogen peroxide in PBS and a specific staining was blocked using Ultra-V Block (Immunologic, VWR International, Radnor, PA). The following primary antibodies were diluted in normal antibody diluent (KliniPath, VWR International, Radnor, PA), applied on sections and incubated overnight in a humidified chamber at 4 °C; anti-CD31 antibody (ab28364, Abcam, Cambridge, UK, 1:400), anti-CD34 Class II antibody (M7165, Dako/Agilent, Santa Clara, CA, 1:1000), anti-VEGFR2 D5B1 antibody (9698S, Cell Signaling, Danvers, MA, 1:500). BrightVision + post antibody block was applied on the sections for 20 min at room temperature followed by secondary antibody BrightVision Poly-HRP-anti Ms/Rb IgG (both Immunologic) for 30 min at room temperature. Staining was developed using Bright-DAB (Immunologic) and sections were dehydrated in a series of ethanol and xylene and mounted in Pertex mounting medium (HistoLab, Askim, Sweden). Quantification of immunohistochemical staining was performed using ImageJ software (Bethesda, MD). Hematoxylin and DAB staining was separated using the color deconvolution plugin. The same threshold was set for each image and the region of interest was selected using the ROI manager tool. DAB or hematoxylin staining was analyzed as percentage of area.

### Quantitative RT-PCR

RNA was isolated (Bioline, London, UK) and cDNA was synthesized using superscript III (Invitrogen, Waltham, MA). Quantitative RT-PCR (qRT-PCR) was performed according to manufacturer’s instructions using SYBR green and a Lightcycler LC480II (Roche, Basel, Switzerland). Transcript levels were calculated with the comparative threshold cycle method and subsequently normalized to *B2M*. Primer sequences: *B2M*-forward 5′-CTTCAGTCGTCAGCATGG, reverse 5′-GTTCTTCAGCATTTGGATTTC; *SPARC*-forward 5′-CCAGGCAAAGGAGAAAGAAG, reverse 5′-TTCAGACCGCCAGAACTCTT.

### Statistical analyzes

Statistical analyzes were carried out using GraphPad Prism 6 (GraphPad Software Inc., San Diego CA). Statistical differences for two groups were evaluated using two-sided unpaired *t*-tests. *In vivo* survival analysis was performed using Log-rank (Mantel–Cox) test and survival rates were expressed using Kaplan–Meier curves. A value of *p* < .05 was considered statistically significant.

## Results

### Pharmacokinetics of nab-paclitaxel after intraperitoneal administration

**Figure 1.**
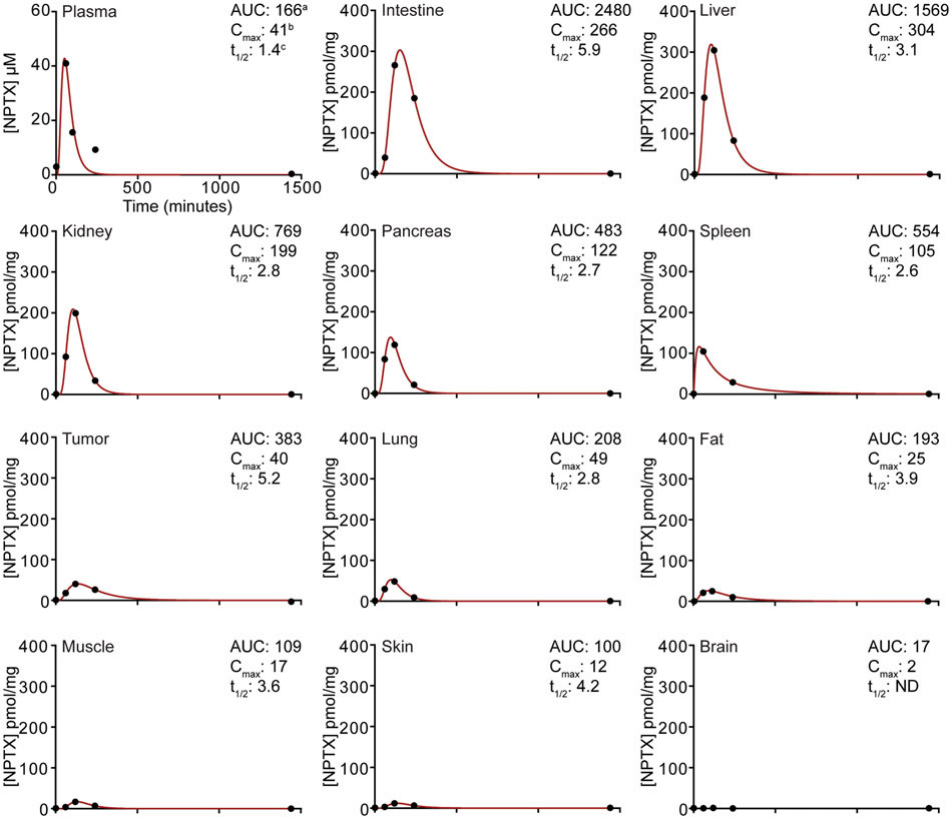
Pharmacokinetics of *nab*-paclitaxel after intraperitoneal administration. Nude mice bearing subcutaneous EAC-derived xenografts of ∼800 mm^3^ received a single bolus of 120 mg/kg NPTX intraperitoneally and at designated time points plasma and tissue samples were collected. NPTX concentration was determined using LC–MS/MS–MS. ^a^AUC values are reported in µM h (plasma) or pmol/mg h (tissues) and represent AUC of 0–24 h post-injection. ^b^*C*_max_ values are reported in µM (plasma) or pmol/mg (tissues) and represent the sample taken at 60 (plasma) and 120 (tissues) min after NPTX injection. ^c^Half-life (*t*_1/2_) is expressed in hours.

To assess the pharmacokinetics of NPTX in plasma, normal tissues, and tumor after i.p. administration, nude mice bearing subcutaneous EAC-derived xenografts of ∼800 mm^3^ received a single bolus of 120 mg/kg NPTX. Subsequently, the concentration of NPTX in plasma and tissue/tumor was measured at designated time intervals using LC–MS/MS analysis (Figure 1).

NPTX concentration reached its maximum concentration (*T*_max_) in plasma 1 h post-injection, after which the concentration decreased rapidly with a half-life (*t*_1/2_) of 1.4 h. Also in normal tissues NPTX was rapidly absorbed with a *T*_max_ of 2 h post-injection in all tissues except for brain, where no NPTX was detected likely due to the blood–brain barrier. At 24 h after administration, NPTX could not be detected in the plasma or tissue suggesting complete elimination.

Area under the curve (AUC) and maximum concentration (*C*_max_) values were highest in intraperitoneal tissues (i.e. intestine, liver, kidney, pancreas, and spleen) with the intestine showing the largest NPTX exposure over time (2480 pmol/mg h, Figure 1). Interestingly, tumors had a 2–4-fold higher AUC value compared to surrounding tissues such as muscle, fat, and skin (383 pmol/mg h vs. 100–193 pmol/mg h, Figure 1). This was due to a longer *t*_1/2_ value. In conclusion, these results demonstrate that i.p. administration of NPTX can be used to determine its uptake in subcutaneously grafted tumors.

### DC101 delays tumor growth and impairs vascularization of tumors and intraperitoneal organs

**Figure 2.**
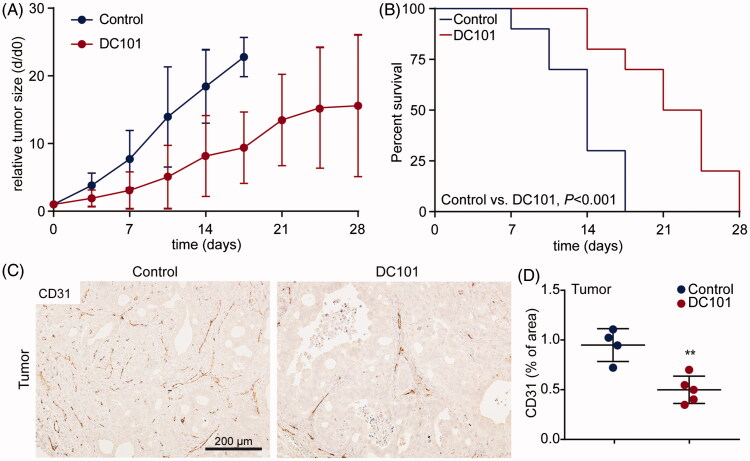
DC101 therapy delays tumor growth and severely impairs vascularization in EAC tumors. (A) Nude mice bearing subcutaneous EAC-derived xenografts of 50–75 mm^3^ were treated with 40 mg/kg DC101 or PBS control therapy twice a week for four weeks. Tumor size was normalized against tumor size at the start of the treatment (day 0). (B) Percent survival of mice after DC101 or control therapy expressed using Kaplan–Meier curves. Log-rank (Mantel–Cox) test was used to determine statistical significance. (C) DC101 treated and control tumors were immunohistochemically stained for CD31 antibody to determine vascularization. (D) Quantification of CD31 staining of tumor sections using ImageJ software as percentage of area. *Line graphs and scatter dot plots* show the mean ± SD, *n* = 5 per group. **p < .01 was determined by two-sided unpaired *t*-tests and analyzed against untreated control.

As NPTX is combined with ramucirumab for second-line treatment of patients with AEG, we assessed the systemic effects of DC101 by studying the vascular changes in tumor and non-tumor tissues and how these affect NPTX uptake, delivery, and elimination. First, effects of one month DC101 therapy were studied in EAC xenograft models. When tumors reached 50–75 mm^3^, DC101 or control treatment was started. Mice received 8 injections of DC101 (40 mg/kg) over the course of 4 weeks. As expected, tumor growth was significantly delayed in mice that received DC101 (Figure 2(A)). Also, survival of animals was prolonged in these animals compared to control (3.2 ± 1.6 weeks (95% CI: 0.7–3.9), and 2 ± 0.6 weeks (95% CI: 0.3–1.5) respectively, Figure 2(B)). To determine if the observed growth delay was associated with vascular regression, tumors were stained for the endothelial cell marker CD31. DC101-treated tumors indeed demonstrated significantly less CD31 positive cells compared to control tumors (Figure 2(C) and (D)).

**Figure 3.**
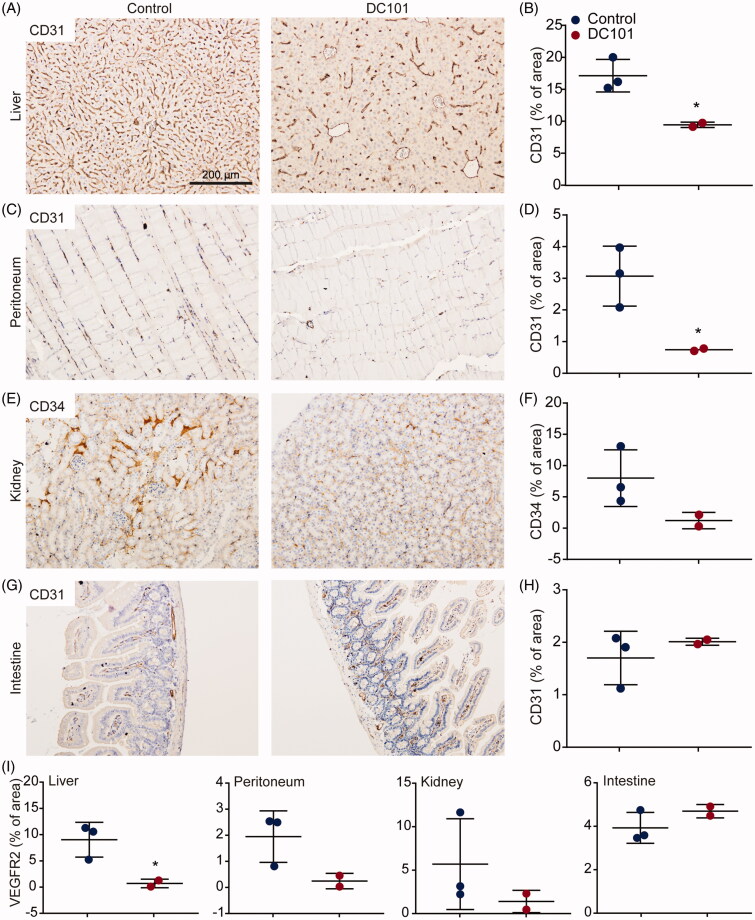
DC101 therapy has systemic off-target effects by reducing vascularization in intraperitoneal organs. Nude mice were treated with 40 mg/kg DC101 or PBS control therapy twice a week for four weeks and changes in vascularization were detected using CD31 or CD34 immunohistochemical stainings on (A) liver, (C) peritoneum, (E) kidney, and (G) intestine tissue sections. CD31 and CD34 stainings in (B) liver, (D) peritoneum, (F) kidney, and (H) intestine were quantified using ImageJ software as percentage of area. (I) Tissue sections of intraperitoneal organs were immunohistochemically stained for VEGFR2 and quantified using ImageJ software as percentage of area. *Scatter dot plots* show the mean ± SD, *n* = 3 per group. *p < .05 were determined by two-sided unpaired *t*-tests and analyzed against untreated control.

Although the anti-tumor effects of angiogenesis inhibition have been extensively studied, to our knowledge, no studies have demonstrated the effects of DC101 therapy on intraperitoneally located healthy tissues. Therefore, we collected the liver, peritoneum, kidney, and intestine of DC101-treated mice and stained these for CD31 to assess changes in vascularization. Since CD31 also stained other structures in kidney, CD34 was used as an endothelial marker in kidney. This revealed that the number of vessels was reduced in the liver, peritoneum, and kidney following DC101 treatment (Figure 3(A)–(F)). In contrast, CD31 positive vasculature was not different in the intestine of DC101-treated mice (Figure 3(G) and (H)). These data imply that DC101 has strong effects in the majority of the intraperitoneally situated tissues.

To confirm that vascular regression was indeed driven by VEGFR2 inhibition, tissues were immunohistochemically stained for VEGFR2 protein expression. Liver, peritoneum, and kidney all showed decreased expression of VEGFR2 following DC101 therapy (Figure 3(I)). VEGFR2 expression in intestine was not altered, in accordance with the CD31 staining (cf. Figure 3(G)).

### Angiogenesis inhibition impairs the intratumoral delivery of intraperitoneally injected nab-paclitaxel

**Figure 4.**
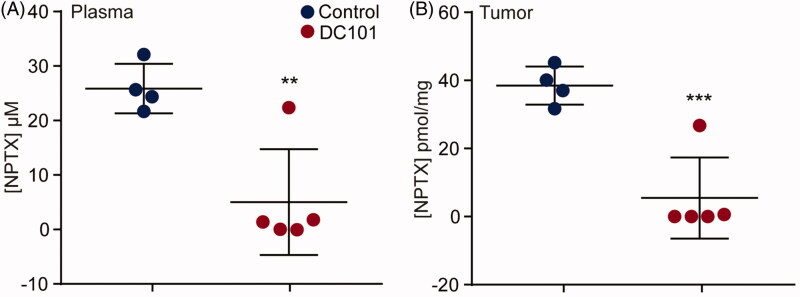
Intratumoral delivery of intraperitoneally injected *nab*-paclitaxel is strongly reduced after angiogenesis inhibition. (A) Nude mice bearing subcutaneous EAC-derived xenografts of 50–75 mm^3^ were treated with 40 mg/kg DC101 or PBS control therapy twice a week for four weeks followed by an intraperitoneal injection of 120 mg/kg NPTX. NPTX concentration in the blood plasma was determined two hours after i.p. administration of NPTX by LC–MS/MS analysis. (B) Of the same mice, subcutaneous tumors were collected, homogenized in normal saline and NPTX concentration was determined by LC–MS/MS analysis. *Scatter dot plots* show the mean ± SD, *n* = 5 per group. **p < .01, ***p < .001 were determined by two-sided unpaired *t*-tests and analyzed against untreated control.

Based on our finding that DC101 impedes vascularization in both tumor and intraperitoneal tissues, we hypothesized that DC101 treatment could alter the uptake of intraperitoneally administered cytotoxic agents and ultimately affect intratumoral therapy delivery. Tumor-bearing mice (50–75 mm^3^) received eight injections over the course of four weeks of DC101 or control therapy and three days after the last injection of DC101 or PBS, a single bolus of 120 mg/kg of weight NPTX was administered via intraperitoneal injection. Two hours after NPTX injection, blood plasma and tumor samples were collected and NPTX concentrations were measured with LC–MS/MS (Figure 4). This revealed that after DC101 therapy there was significantly less NPTX present in tumor tissue samples compared to control (Figure 4(B)). Previous studies have suggested that the enhanced tumor uptake of NPTX is mediated by the binding of albumin to SPARC expressed on stromal cells (Kiessling et al., 2002; Knauer et al., 2009; Von Hoff et al., 2011). However, qRT-PCR analysis revealed that stromal SPARC was not altered following DC101 therapy suggesting that the decreased intratumoral uptake of NTPX is not SPARC-mediated (Supplementary Figure 1). Alternatively, the reduced intratumoral NPTX concentration could be due to the observed decreased tumor vascularization but the low plasma concentrations of NPTX suggest that the NPTX never reached the circulation. These results could imply that DC101 therapy impedes the uptake of NPTX from the peritoneal cavity. Conversely, the low NPTX plasma concentrations could be explained by an accelerated elimination of NPTX from the intraperitoneal cavity following DC101 therapy.

### Elimination routes of intraperitoneally administered nab-paclitaxel are affected by angiogenesis inhibition

**Figure 5.**
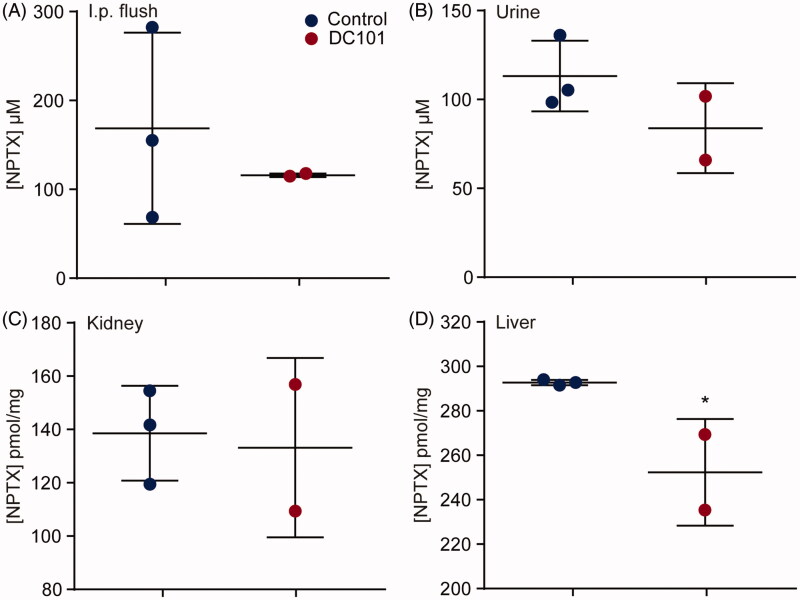
Elimination routes of intraperitoneally administered *nab*-paclitaxel are affected by angiogenesis inhibition. Nude mice were treated with 40 mg/kg DC101 or PBS control therapy twice a week for four weeks followed by an intraperitoneal injection of 120 mg/kg NPTX. Of all mice, the (A) intraperitoneal cavity lavage (B) urine, (C) liver, and (D) kidney were collected and NPTX concentrations were determined using LC–MS/MS. *Scatter dot plots* show the mean ± SD, *n* = 3 per group. *p < .05 was determined by two-sided unpaired *t*-tests and analyzed against untreated control.

NPTX is predominantly eliminated *via* hepatic metabolism and biliary excretion, and only a small amount is excreted via urine. To determine whether DC101 therapy affects either the uptake of NPTX from the peritoneal cavity or elimination of NPTX from the body, we measured the concentration of NPTX in these systems. Mice received DC101 or control therapy and three days after the last injection, 120 mg/kg of weight NPTX was injected intraperitoneally. Two hours later, mice were sacrificed and NPTX concentrations were measured in the peritoneum (by flushing the intraperitoneal cavity), urine, liver, and kidney using LC–MS/MS. Our results showed that intraperitoneal cavity lavage, urine, and kidney NPTX concentrations were not altered after DC101 therapy (Figure 5(A)–(C)). Concentration of NPTX was decreased in the liver at 2 h after intraperitoneal injection (Figure 5(D)). These results imply that the lowered NPTX plasma levels following VEGFR2 inhibition cannot be explained by an accumulation in the peritoneal cavity, or changes in renal clearance following VEGFR2 inhibition. However, elimination *via* the hepatic system might be affected since less NPTX is present in the liver.

### Nab-paclitaxel elimination is associated with altered intestinal mucosa

We have demonstrated that lower concentrations of NPTX were present in the liver after DC101 therapy, indicating reduced or accelerated elimination *via* the hepatic system, while its renal clearance remained unchanged and there was no accumulation of NPTX in the peritoneum. Given the low blood plasma and tumor tissue NPTX concentrations, we hypothesized that another route of elimination is responsible for NPTX clearance after DC101 therapy. Based on the pharmacokinetics of NPTX after intraperitoneal administration, exposure is highest in intestine suggesting that also in control animals a high amount of NPTX is eliminated *via* the feces (Figure 1). However, this is most likely not achieved via the hepatic system followed by secretion into the bile and feces as significantly lower levels of NPTX were detected in the liver after DC101 therapy (Figure 5(C)). Therefore, we assessed whether DC101 therapy induced histological changes in the intestine that could explain an enhanced elimination of NPTX.

**Figure 6.**
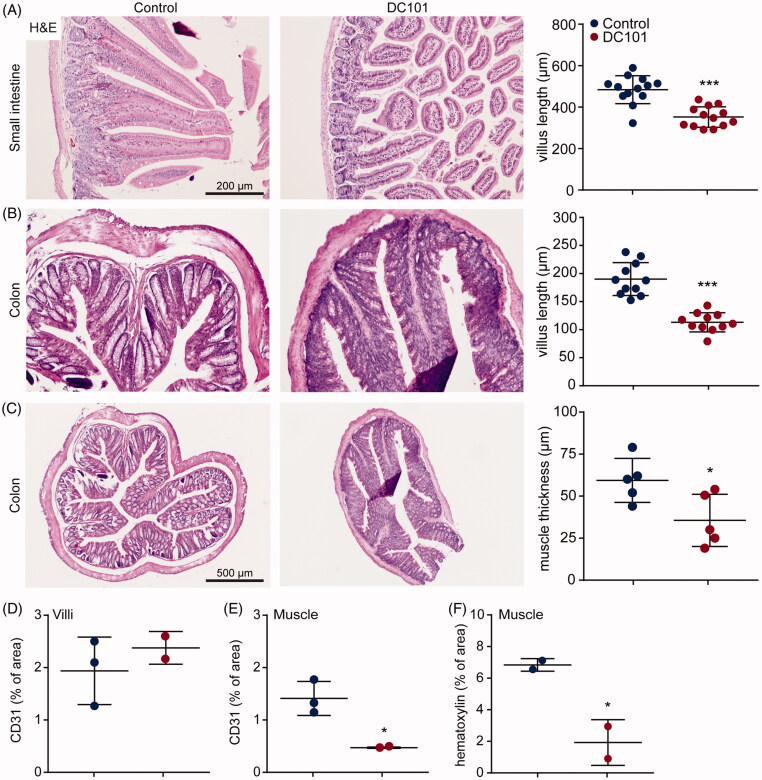
*Nab*-paclitaxel elimination is associated with altered intestinal mucosa. (A) Nude mice were treated with 40 mg/kg DC101 or PBS control therapy twice a week for four weeks and intestine were collected for histological analysis. Small intestine was stained for H&E and villus length was measured using ImageJ software, 4–5 villi were measured per section. (B) Colon was stained for H&E and villus length was measured using ImageJ software, 2–3 villi were measured per section. (C) Thickness of the muscle layer lining the colon was measured using ImageJ software, 1–2 areas per section were measured for muscle thickness. (D) Villi of the intestine were immunohistochemically stained for CD31 and quantified using ImageJ as percentage of area. (E) The muscle layer lining the colon was immunohistochemically stained for CD31 and quantified using ImageJ as percentage of area. (F) The amount of nuclei (hematoxylin) in the muscle layer lining the colon was quantified using ImageJ and depicted as percentage of area. *Scatter dot plots* show the mean ± SD, *n* = 3 per group. *p < .05, ***p < .001 was determined by two-sided unpaired *t*-tests and analyzed against untreated control.

Intestines of mice which received DC101 or control therapy were collected and histological assessment revealed that in both small intestine and colon, villus length was significantly decreased upon DC101 therapy (Figure 6(A) and (B)). Quantification of CD31 positive cells showed that villus vascularization was not affected by DC101 therapy, suggesting that these histological changes are not due to vascular regression in the villi (Figure 6(D)). Also, the muscle layer lining the villi of the colon was significantly reduced in thickness compared to control (Figure 6(C)). To determine whether this was the result of a reduction in size (i.e. atrophy) or reduction in the amount of muscle cells, we quantified the number of nuclei per area and showed a significant decrease in the number of muscle cells (Figure 6(F)). In addition, the number of vessels in the muscle layer was significantly decreased (Figure 6(E)). These results suggest that DC101 therapy hampers the delivery of nutrients via vascular regression, which negatively impacts on the muscle cells lining the colon. Combined with the reduced villus length, overall thickness of the intestinal wall is strongly reduced after VEGFR2 inhibition and this could explain why NPTX is eliminated faster *via* the intestine after intraperitoneal administration.

## Discussion

Angiogenesis inhibition is a widely used therapeutic approach against various solid tumors. The VEGFR2 inhibitor ramucirumab has been approved by the FDA for the treatment of advanced gastric cancer and gastroesophageal junction cancer in 2014 either as monotherapy or in combination with paclitaxel (Fuchs et al., 2014; Wilke et al., 2014). However, ramucirumab only modestly improves median overall survival in these patients, and mechanistic insights into target and off-target effects are needed to determine if, and how, this can be improved. Despite antiangiogenic drugs being widely used in clinic, little is known about the effects of these drugs on healthy tissues and how this might affect the uptake and pharmacokinetics of chemotherapy. In the present study, we aimed to elucidate these issues. We showed that VEGFR2 inhibition reduced vasculaturization in healthy organs and, as a consequence, altered the pharmacokinetics of intraperitoneally administered NPTX. This resulted in reduced intratumoral delivery of chemotherapy in a mouse model for EAC.

Preclinical studies investigating the effects of antiangiogenic drugs on healthy tissues have shown that especially endocrine organs such as the thyroid are affected (Kamba, 2005; Yang et al., 2013). We focused on the effects of DC101 therapy on tissues located intraperitoneally and showed substantial vascular regression in the liver, peritoneum and kidney. This is in agreement with a previous study demonstrating that anti-VEGFR2 therapy significantly decreased vessel area per field in both liver and kidney (Kamba, 2005). While preclinical studies administer antiangiogenic drugs intraperitoneally, in patients these drugs are given *via* i.v. infusion. However, frequently occurring adverse effects in patients such as proteinuria and liver test abnormalities suggests the effects of antiangiogenic drugs on healthy tissues are systemic and are not determined by the route of administration (Wang et al., 2016). As we found that intraperitoneally located organs were affected by DC101 therapy, we hypothesized this could affect uptake and delivery of NPTX after i.p. administration. Recent studies demonstrate the increased antitumor activity of NPTX compared to conventional solvent-based paclitaxel (sb-PTX) with equitoxic doses (Kinoshita et al., 2014). Therefore, it is likely that in future clinical practice NPTX will be the preferred regimen of choice (over sb-PTX) and we decided to study NPTX in our experiments. To our knowledge, no studies have investigated the pharmacokinetics of NPTX in mice in a wide range of tissues after i.p. administration. We demonstrated that the highest exposure to NPTX was measured in intraperitoneally located organs and *C*_max_ was reached two hours after administration. Compared to a study performed by Gustafson and colleagues, who investigated the pharmacokinetics of sb-PTX after i.v. injection, NPTX demonstrated on average twice as high *C*_max_ and AUC values in plasma and tissues after i.p. administration while *t*_1/2_ remained the same (Gustafson et al., 2005). This corresponds to studies demonstrating more efficient transport of NPTX across endothelial cells compared to sb-PTX, in which albumin results in an advantageous pharmacokinetic profile (Yardley, 2013; Chen et al., 2015). Interestingly, this was not the case for intestinal tissue, where *t*_1/2_ was increased after i.p. administration of NPTX resulting in an almost four times higher AUC compared to i.v. administered sb-PTX (Gustafson et al., 2005). This was supported by our observations in mice, finding severe diarrhea quickly after i.p. injection of NPTX suggesting that i.p. administered NTPX has a relatively high affinity for the intestine. Repeated intraperitoneal injection is therefore not advocated given the severe diarrhea that developed at this dose level. Also, we found that tumor tissue contained 2–4-fold higher NPTX AUC value compared to surrounding tissues (i.e. muscle, fat, skin). This once again suggests that NPTX concentration is not strictly related to the location of the tissue, but rather defined by either the affinity for or elimination rate in specific tissues.

It is assumed that high-dose or long-term anti-angiogenic therapy prunes vasculature, thus increasing hypoxia and hampering drug delivery (Franco et al., 2006; Riesterer, 2006; Robert et al., 2011; Van Cutsem et al., 2011; Huang et al., 2012). However, to our knowledge we are the first to formally demonstrate that after one month of angiogenesis inhibition, delivery of chemotherapeutic agents to plasma and tumor tissue is severely decreased following intraperitoneal administration. The decrease in plasma NPTX concentration following DC101 therapy suggests that either the uptake or elimination of NPTX from the peritoneal cavity is altered, resulting in reduced intratumoral NPTX delivery. This is of great relevance since in preclinical studies the majority of therapies is administered i.p. Also, recent studies recommend intraperitoneal administration of paclitaxel in patients with advanced gastric cancer (Soma et al., 2009; Kinoshita et al., 2014).

In an attempt to explain the decreased tumor and plasma NPTX concentrations, we found that DC101 therapy altered the elimination of NPTX, which was associated with strongly decreased hepatic levels of NPTX. This implies either a reduced or accelerated hepatic clearance following DC101 therapy. Based on the decrease in VEGFR2 expression in the liver following DC101 therapy and consecutive vascular regression, we conclude that the liver is strongly dependent on the VEGF/VEGFR2 signaling axis for angiogenesis. This could explain the lower hepatic levels of NPTX following DC101 therapy and suggest reduced uptake of NPTX *via* the liver. We also revealed that the intestinal mucosa is altered upon DC101 therapy, with a significant decrease in villus length and thickness of the muscle layer. Other pathologies which are characterized by shortening of villus length and mucosal atrophy, such as ulcerative colitis or inflammatory bowel disease, demonstrate an epithelial barrier dysfunction resulting in an easily penetrable mucosa and chronic intestinal inflammation (Bayless & Hanauer, 2011; Antoni, 2014; Johansson et al., 2014). This can explain the very frequently occurring adverse events in patients receiving ramucirumab, such as diarrhea, mucosal inflammation and the increased risk of gastrointestinal perforations (Fala, 2015; Wang et al., 2016). This also implies that, upon DC101 therapy, intestinal mucosa becomes more permeable for intraperitoneally administered agents. This could be a novel mechanism by which NPTX is discarded after i.p. injection resulting in lower concentrations in tissues including liver, plasma, and tumor. Since both elimination *via* the hepatic system and the intestinal mucosa eventually lead to elimination *via* the feces, measuring the fecal NPTX concentrations cannot discriminate between changes in one system from the other. Lastly, these results suggest that anti-angiogenic therapy could make the intestinal mucosa more susceptible to intraperitoneally administered cytotoxic agents and could therefore be an interesting therapeutic strategy for colorectal cancer. Future research should elucidate the exact mechanism driving intestinal mucosa changes following VEGFR2 inhibition and how these affect uptake and elimination of intraperitoneally injected cytotoxic agents.

In conclusion, our preclinical findings reveal that VEGFR2 inhibition results in systemic vascular regression thereby interfering with the uptake, delivery, and elimination of intraperitoneally administered *nab*-paclitaxel. These data could clarify adverse events occurring in clinic, but also imply that more understanding is necessary in the combined use of anti-angiogenesis and cytotoxic agents for both preclinical and clinical use.

## Supplementary Material

IDRD_Steins_et_al_Supplement_Content.tif
