# Supplementary figures and images for: Systemic effects of angiogenesis inhibition alter pharmacokinetics and intratumoral delivery of nab-paclitaxel
Source: Drug Deliv. 2017 Nov 24;24(1):1801–10. doi: 10.1080/10717544.2017.1406559 (PMC8241153; doi:10.1080/10717544.2017.1406559)

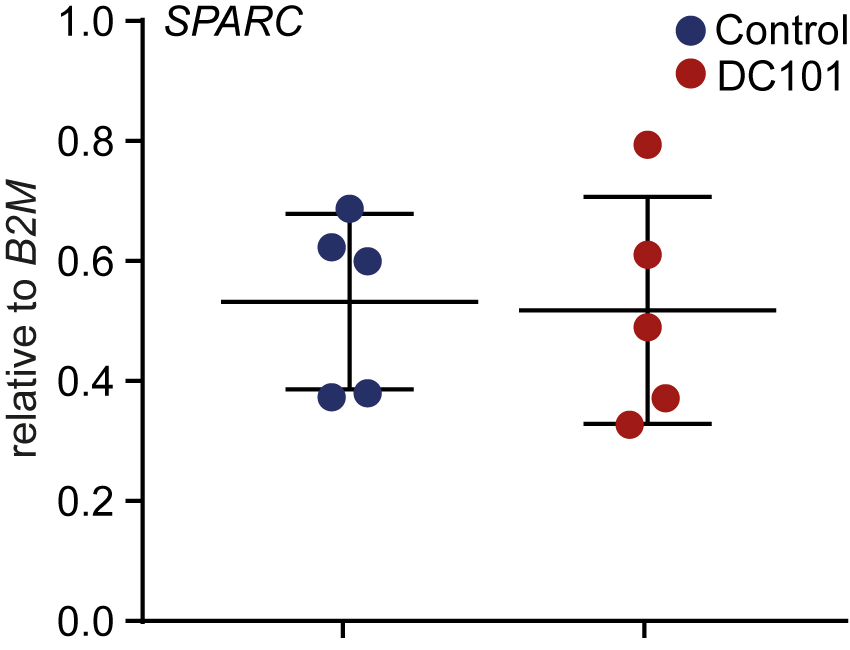

Supplement: IDRD_Steins_et_al_Supplement_Content.tif [file IDRD_A_1406559_SM2996.tif]
